# Comparison of systematic triage with clinical assessment in prediction of short-term mortality

**DOI:** 10.1186/1757-7241-23-S1-A15

**Published:** 2015-07-16

**Authors:** Anne K S Iversen, Jakob L Forberg, Morten Schou, Lars S Rasmussen, Lars Køber, Kasper Iversen

**Affiliations:** 1 Department of Cardiology and Endocrinology, Hospital Of Northern Zealand, Hillerød Hospital, Hillerød, Denmark; 2 Department of Emergency Medicine, Hospital Of Northern Zealand, Hillerød Hospital, Hillerød, Denmark; 3 Department of Cardiology, Herlev Hospital, University of Copenhagen, Copenhagen, Denmark; 4 Department of Anaesthesia, Centre Of Head and Orthopaedics, Rigshospitalet, University of Copenhagen, Copenhagen, Denmark; 5 Department of Cardiology, Rigshospitalet, University of Copenhagen, Copenhagen, Denmark

## Background

Prior to introduction of systematic triage, patients were prioritized in Emergency Departments based on clinical assessment. Validation of systematic triage is sparse and in this study we compared the systematic triage tool Danish Emergency Process Triage (DEPT) with a quick clinical assessment by inexperienced hospital staff as markers of short-term mortality.

## Method

A prospective cohort study was conducted at Hillerød University Hospital. All patients admitted to the Emergency Department (ED) from September 2013 to December 2013 were included. Triage was performed by a trained nurse using the ED's standard triage tool, DEPT, and patients were categorized as green (not urgent), yellow, orange or red (most urgent). A phlebotomist performed a quick clinical assessment (eyeball triage) to do the same categorisation but only based on a look at the patient and the main complaint. The primary endpoint was 30-day mortality.

## Results

A total of 6,383 admissions (5,568 patients) were included. DEPT triage was performed for 6,290 (98.5%) and eyeball triage for 6,382 (>99.9%) of the admissions. The DEPT triage respective eyeball triage characterized 32.3% vs. 37.3% of the patients as green, 39.0% vs. 44.6% as yellow, 26.7% vs. 16.2% as orange and 0.6% vs. 1.8% as red. Agreement described as Kappa was 0.05. Receiver operation characteristics (ROC) analysis of the prognostic value of DEPT and eyeball triage in relation to 30-day mortality showed that the area under the curve for DEPT triage was 0.62 (95% CI, 0.58-0.65) and 0.73 (95% CI, 0.70-0.76) for eyeball triage, p < 0.01. Analysis of 30-day mortality showed that the hazard ratio for patients categorized as yellow with DEPT triage was 1.7, orange 2.6, and red 19.1 (green is reference). The corresponding hazard ratios for eyeball triage were 2.4, 7.9, and 27.5. The negative predictive value of being green or yellow in relation to 30-day mortality was 97.6% (97.2-98.0) for eyeball triage and 96.8% (96.2-97.3) for DEPT, p < 0.01.

## Conclusion

Agreement between DEPT and eyeball triage was poor. The clinical assessment by inexperienced hospital staff was a significant better prognostic marker with regards to 30-day mortality risk. This observation questions the value of systematic triage as used today.

